# Supplementary material for: System-wide analysis of RNA and protein subcellular localization dynamics
Source: Nat Methods. 2023 Nov 30;21(1):60–71. doi: 10.1038/s41592-023-02101-9 (PMC10776395; doi:10.1038/s41592-023-02101-9)
Supplement: Supplementary file 1 — Supplementary Figs. 1–7 and information on Supplementary Tables 1–4. [file 41592_2023_2101_MOESM1_ESM.pdf]

nature methods

Article https://doi.org/10.1038/s41592-023-02101-9

System-wide analysis of RNA and protein subcellular localization dynamics

In the format provided by the authors and unedited

Supplementary figures and information

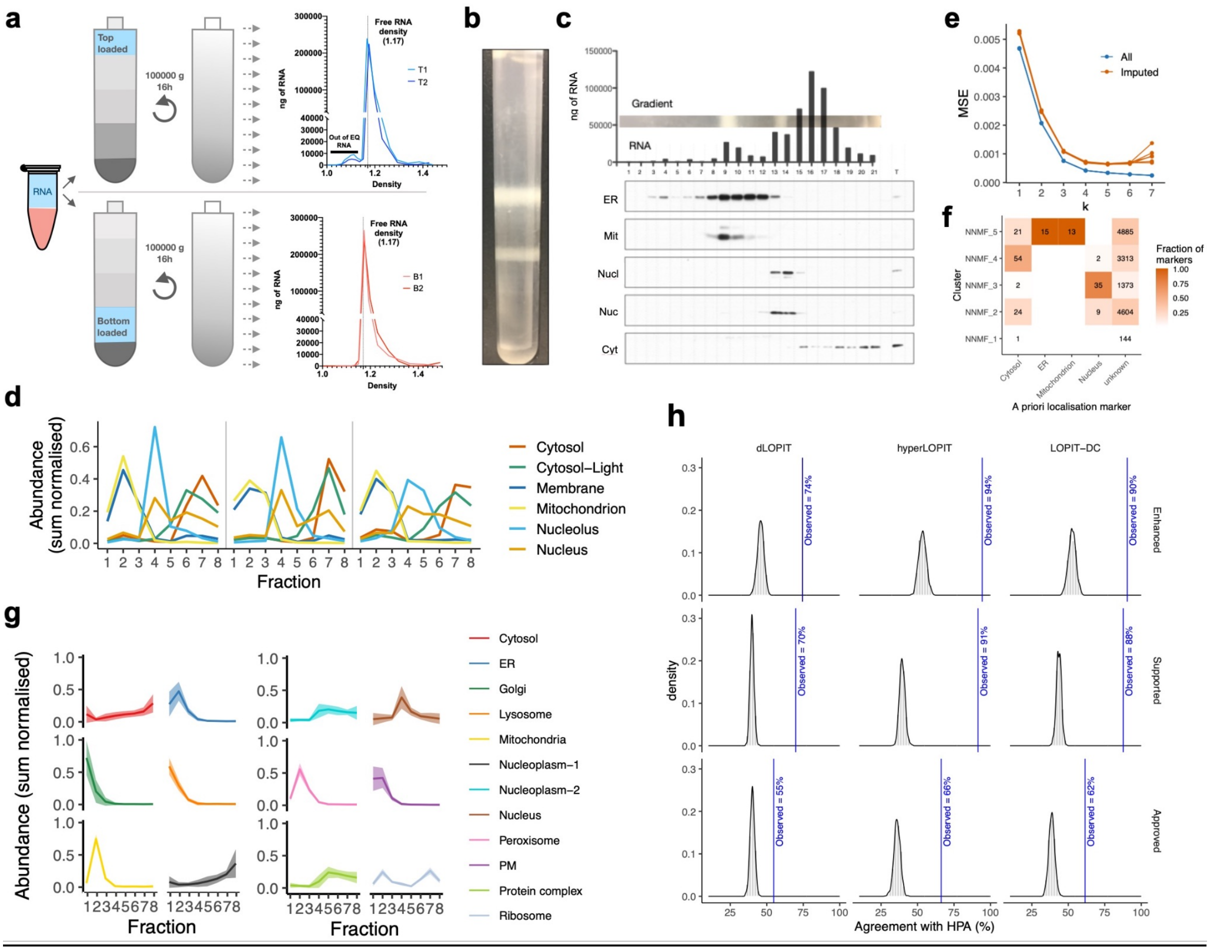

Supplementary figure 1. Simultaneous maps of RNA and protein subcellular localisation
a, Cell-free RNA sedimenting profile along the density gradient. T = top loading (blue), B = bottom loading (red, at 1.17 g/ml). Fractions with density < 1.17 are enriched in organelles. **b**, Representative image of a density-based cell fractionation gradient post centrifugation. **c**, Quantification of RNA (in ng) at the different gradient fractions, and a representative western blot of ER (Calreticulin), mitochondria (Mit, Cytochrome c oxidase subunit 4), nucleolus (Nucl, Fibrillarin), nucleus (Nuc, Histone H3) and cytosolic (b-Actin) compartment markers. Similar results were obtained from 3 independent experiments. **d**, Linear profiles of the indicated RNA markers per replica. **e**, Selection of optimal K for NNMF clustering of gene-level profiles in control condition. MSE = Mean Standard Error. Imputed = data points that were removed and imputed by NNMF. MSE lowest for imputed data points at k=5. **f**, Intersections between highest scoring NNMF cluster and a priori markers, where ‘unknown’ denotes non-markers. **g**, Protein quantification by LC-MS3 and representative protein marker profiles for one replicate. Line and shaded region represents mean +/- one standard error. **h**, Density plots showing null distributions and the observed values for the agreement between LOPIT classifications and Cell Atlas data from the Human Protein Atlas (HPA). Agreement means the LOPIT classification is within the Cell Atlas annotations. Randomisation tests were performed 1000 times for each HPA level and for each LOPIT dataset. In each case the observed values exceeded the 100th percentile of the null distribution. The observed agreement is marked on the density plots in blue.

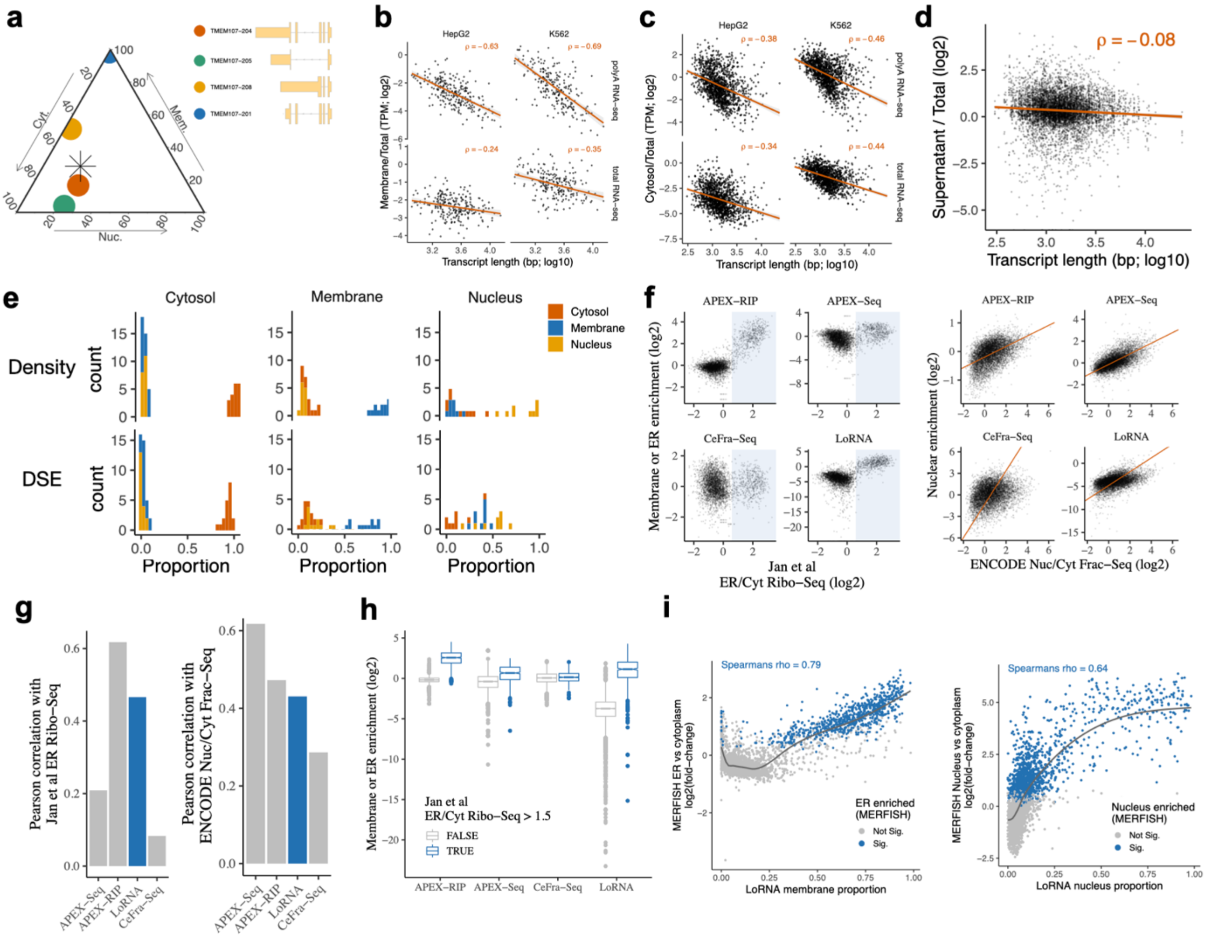

Supplementary figure 2. System-wide quantification of RNA localisation

a, Cytosol, membrane and nucleus proportions for TMEM107 transcript isoforms. Gene-level proportion indicated with asterisk (left). Transcript models (right). **b**, Transcript length vs CeFra-Seq membrane / total for RNAs with > 80% membrane proportion according to equilibrium density centrifugation-based LoRNA. **c**, As per b, except cytosol / total for RNAs with > 80% cytosol proportion **d**, As per b, except differential centrifugation speed-based LoRNA final fraction / total for RNAs with > 80% cytosol proportion. **e**, Proportions for cytosol, membrane and nucleus markers using equilibrium density centrifugation ('Density') and differential sedimentation ('DSE') based cell fractionation. **f**, Comparison of methods for detecting membrane (CeFra-Seq/LoRNA) or ER (APEX-Seq/APEX-RIP) enriched RNAs, against ER Ribo-Seq, and nuclear enrichment (all methods) against nucleus/cytoplasm fractionation RNA-Seq. Shaded area denotes RNAs enriched > 1.5 fold, line represents the total least squares regression. **g**, Pearson correlation vs ER Ribo-Seq and Nuc/Cyt RNA-Seq. Correlation for ER Ribo-Seq was calculated for RNAs with ER/Cyt Ribo-Seq > 1.5 (blue box in f). **h**, Membrane/ER enrichment for RNAs with ER/Cyt Ribo-Seq > 1.5 (n = 605) and RNAs with ratio < 1.5 (n = 3396). Box extends to the 25th and 75th percentiles. Whiskers extend to range, excluding outliers. Outliers are defined as greater than 1.5 x the inter-quartile range from the box. **i,** Comparison between LoRNA proportions and MERFISH ER and Nucleus enrichments. Spearman rank correlation coefficient calculated for RNAs which were significantly enriched in MERFISH.

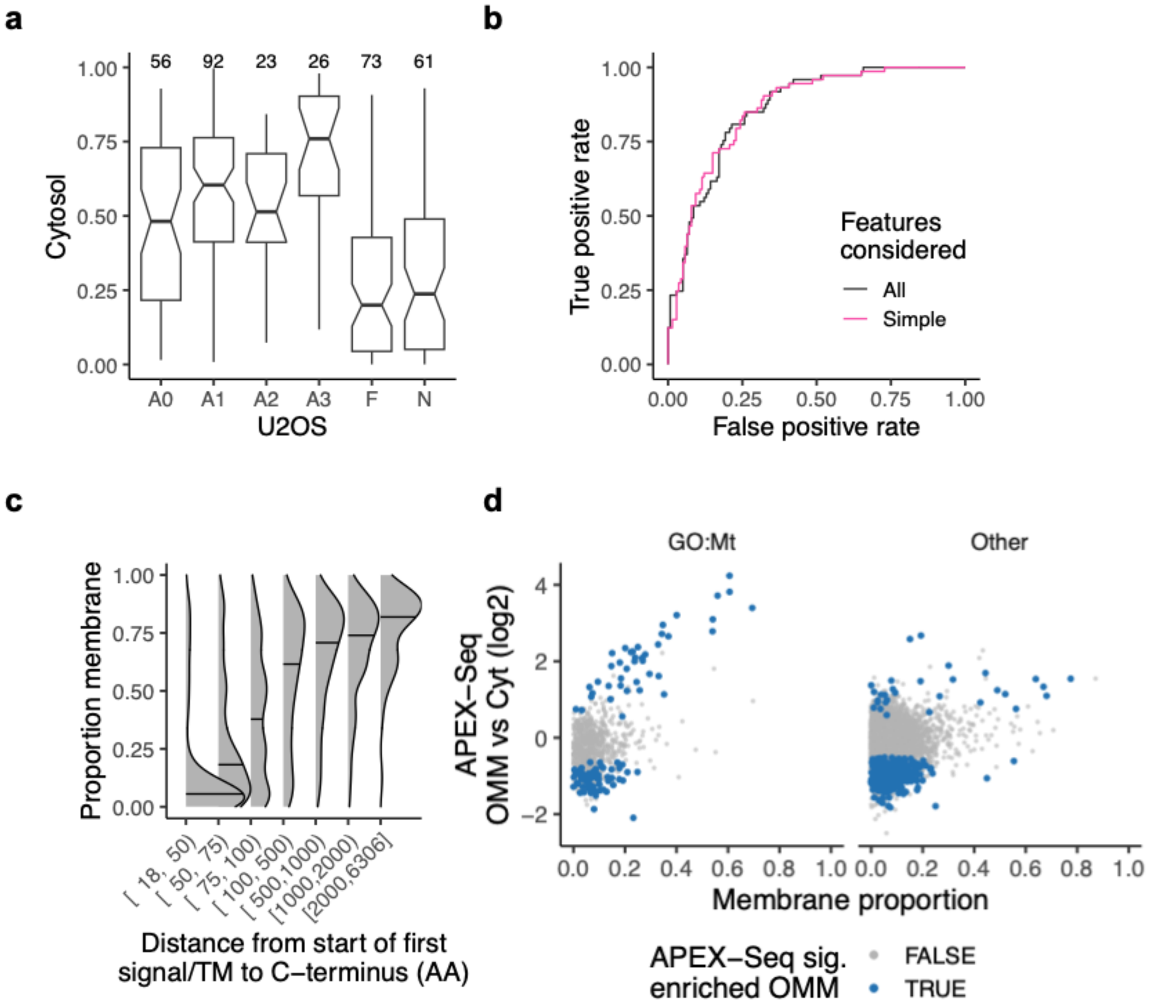

Supplementary figure 3. Features driving RNA localisation

a, Cytosol proportions for lncRNAs annotated by their ribosome association[34]. A0-A3 represent increasingly confident ribosome association. F=Not associated. N=Undetermined. Numbers above boxes indicate the number of lncRNAs. Box extends to the 25th and 75th percentiles. Whiskers extend to range, excluding outliers. Outliers are defined as greater than 1.5 x the inter-quartile range from the box. **b**, Receiver operating characteristic curves for logistic regression models of lncRNA cytosol localisation. All=Lasso regression using all features. Simple=Just using AU content, polyA status and transcript length. **c**, Relationship between the membrane proportion of an mRNA and the distance from the first signal peptide or transmembrane domain to the stop codon of the polypeptide product. **d**, RNAs annotated as encoding mitochondrial proteins in GO and also enriched in APEX-Seq OMM[10] (vs cytosol) have high membrane proportions in LoRNA.

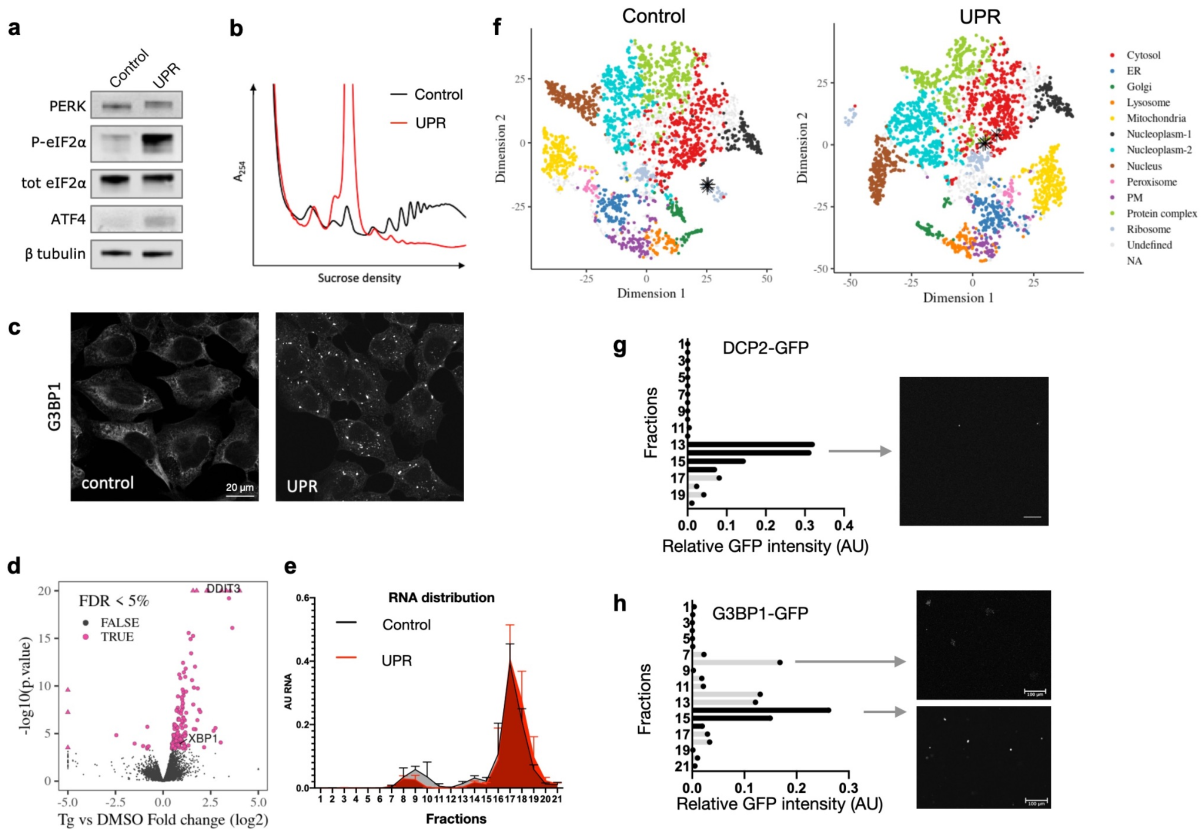

Supplementary figure 4. Transcriptome and proteome subcellular redistribution upon UPR
a, Representative western blot for samples treated with DMSO or TG showing canonical UPR markers. TG treatment induces: a shift in PERK size, consistent with phosphorylation; phosphorylation of eIF2α; increased expression of stress-specific transcription factor ATF4 (n = 3 independent experiments). **b**, Representative polysome profiles in control (DMSO) and UPR (1 h at 250 mM thapsigargin). **c**, Representative Z-projection images of control (DMSO) and UPR-induced (TG 1h) U-2 OS cells immuno-labelled with an antibody for stress granule marker G3BP1 (n = 3 independent experiments). **d**, RNA abundance changes between UPR and control. Significant changes (5% FDR) highlighted. DDIT3/CHOP and XBP1 labelled. P-values derived from Negative Binomial generalised linear model and moderated Wald statistic using DESeq2. **e**, RNA profile along unpooled density gradient fractions in control and UPR conditions. Fractions 1:11 represent light organelles (including mitochondria and ER), fractions 13 and 14 nucleus, and 15 to 21 cytosol (n = 3 per condition, SD indicated by error bars). **f**, t-SNE projections for protein localisation profiles in Control and UPR. The four proteins that relocalise away from the ribosome and towards the cytosol light profile are highlighted with asterisks. Proteins are coloured by their localisation allocation from BANDLE. Proteins with inconsistent localisation allocation are denoted as 'Undefined'. **g**, DCP2-GFP sedimentation profile along unpooled density gradient fractions measured as GFP fluorescence intensity per fractions. Fractions corresponding to the density range of the cytosol light (pooled fraction 6 in Fig 1) in black (left), and representative images of the peaking fraction containing DCP2 granules respectively (right). n=1. Scale bar = 100 µm. **h**, G3BP1-GFP sedimentation profile along unpooled density gradient fractions under UPR stimulation measured as GFP fluorescence intensity per fractions. Fractions corresponding to the density range of the cytosol light in black (left), and representative images of fractions 7 and 14 representing membrane enclosed protein aggregates and G3BP1 containing granules respectively (right). n=1. Scale bar = 100 µm.

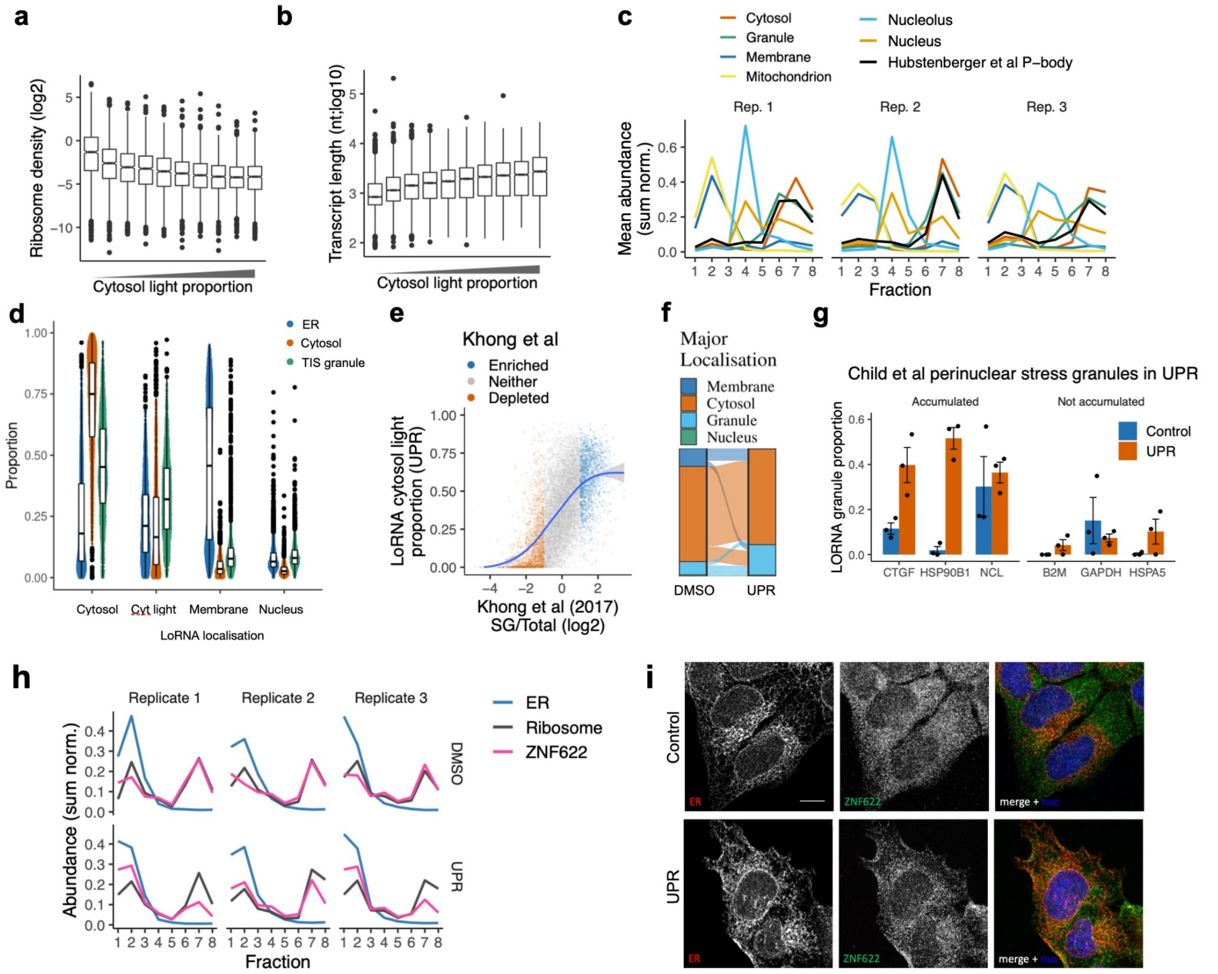

Supplementary figure 5. Analysis of the characteristics driving RNAs to granules

a, Ribosome density for RNAs split into deciles of cytosol light proportion, shown as boxplots. n = 1074 genes for each cytosol light proportion bin. Horizontal line = median. Box extends to the 25th and 75th percentiles. Whiskers extend to range, excluding outliers. Outliers are defined as greater than 1.5 x the interquartile range from the box. **b,** As per (a) for transcript length. n = 3271 transcripts for each cytosol light proportion bin.**c**, Mean profiles for RNA markers (including P-body proteins) across the 3 replicates in control condition. **d**, LoRNA proportions for RNAs classified as cytosolic (CY; n = 1614 genes), canonical rough ER (ER; n = 2170) and TIS granules (TIS; n=1345). Box extends to the 25th and 75th percentiles. Whiskers extend to range, excluding outliers. Outliers are defined as greater than 1.5 x the interquartile range from the box. **e**, Agreement between arsenite-induced SG enrichment and LoRNA cytosol light proportion upon UPR activation. Blue line indicates smoothed fit by LOESS regression. Grey band indicates 95% confidence interval. **f**, Primary localisation of transcripts in control and upon UPR activation. **g**, Granule proportions for RNAs found to accumulate/not accumulate in stress granules upon UPR activation [48]. n = 3 LoRNA experiments. Error bars indicate +/- SE. **h**, Abundance profile of ZNF622 in Control and UPR, with average profiles for ER and ribosome markers. **i**, Representative IF images showing localisation of ZNF622 relative to the ER (calnexin) in control conditions (left panel) and upon UPR activation (right panel; n=2 independent experiments). Scale bar = 10 µm.

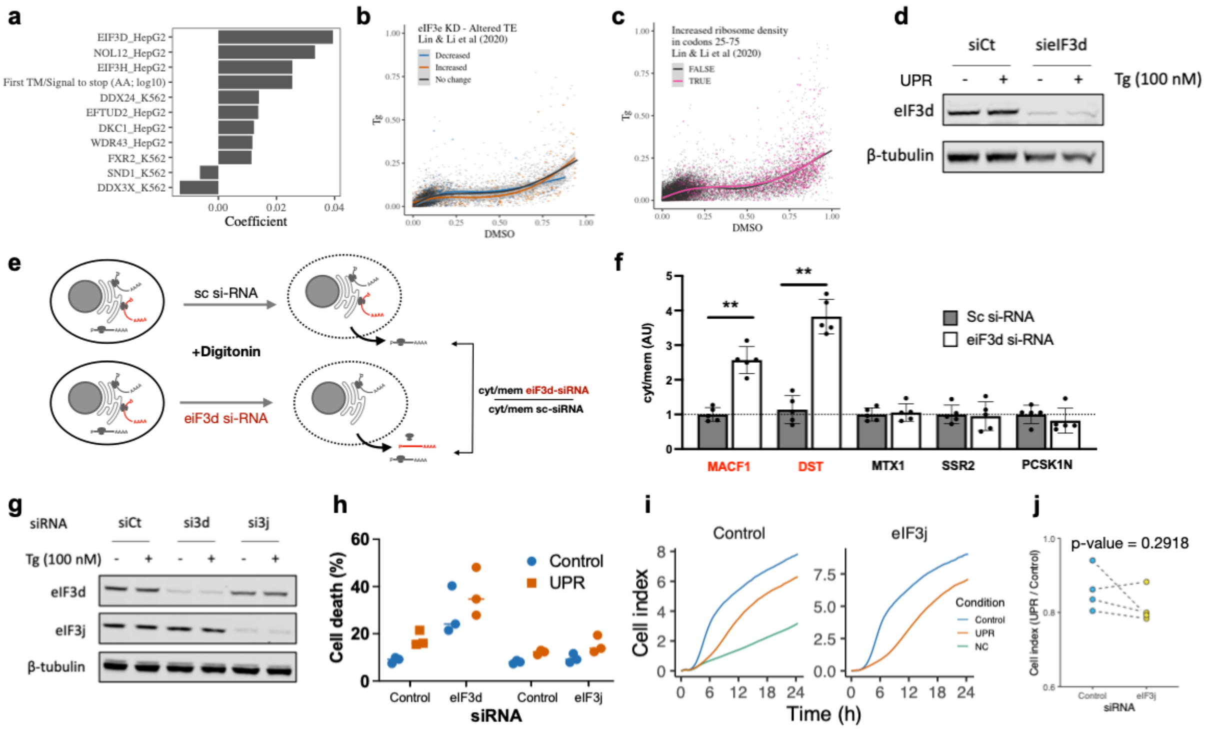

Supplementary figure 6. Analysis of the RNAs retaining membrane association under UPR
a, Coefficients for RBPs which are predictors of GAM residuals for RNAs which encode a signal peptide/TM domain. **b**, **c**, Membrane proportions in control and UPR. R Lines indicate smoothed fit by generalised additive model with cubic regression spline. Grey band indicates 95% confidence interval. RNAs with altered transcriptional efficiency (TE) or increased ribosome binding in codons 25-75 upon eIF3e knockdown[53] are highlighted and appear indistinct from other RNAs. **d**, Representative western blot validating eIF3d knockdown (n=3 independent experiments). **e**, Schematic representation of the cytosolic and membrane associated RNA extraction using digitonin. **f,** Cytosolic and membrane associated RNA extraction using digitonin in control inductions. MTX1, SSR2 and PSK1N are non-eIF3d ER-associated controls. All RNAs are normalised to the MT-ND6 housekeeping transcript. Changes in RNA localisation upon eiF3d siRNA are relative to the RNA localisation in the scrambled si-RNA condition. Error bars represent SD (n=5 independent cytosol and membrane RNA extractions per condition) **P=0.0079 (two-tailed Mann-Whitney U test). **g**, Representative western blot validating eIF3d and eIF3j knockdown. **h**, Quantification of cell death using Annexin V-FITC and Draq7 staining following eIF3d and eIF3j knockdown. Cells were treated with a 1 hour pulse of thapsigargin and allowed to recover for a further 24 hours (n=3 independent experiments). **i**, Representative U-2 OS cell migration time series for eIF3j knock-down and siRNA Control (siCt) cells in control and UPR conditions. **j**, Quantification of cell migration from (i) at 24 h post UPR induction in eIF3j knock-down and siRNA Control cells (n=4 independent experiments). P-value from two-tailed paired t-test.

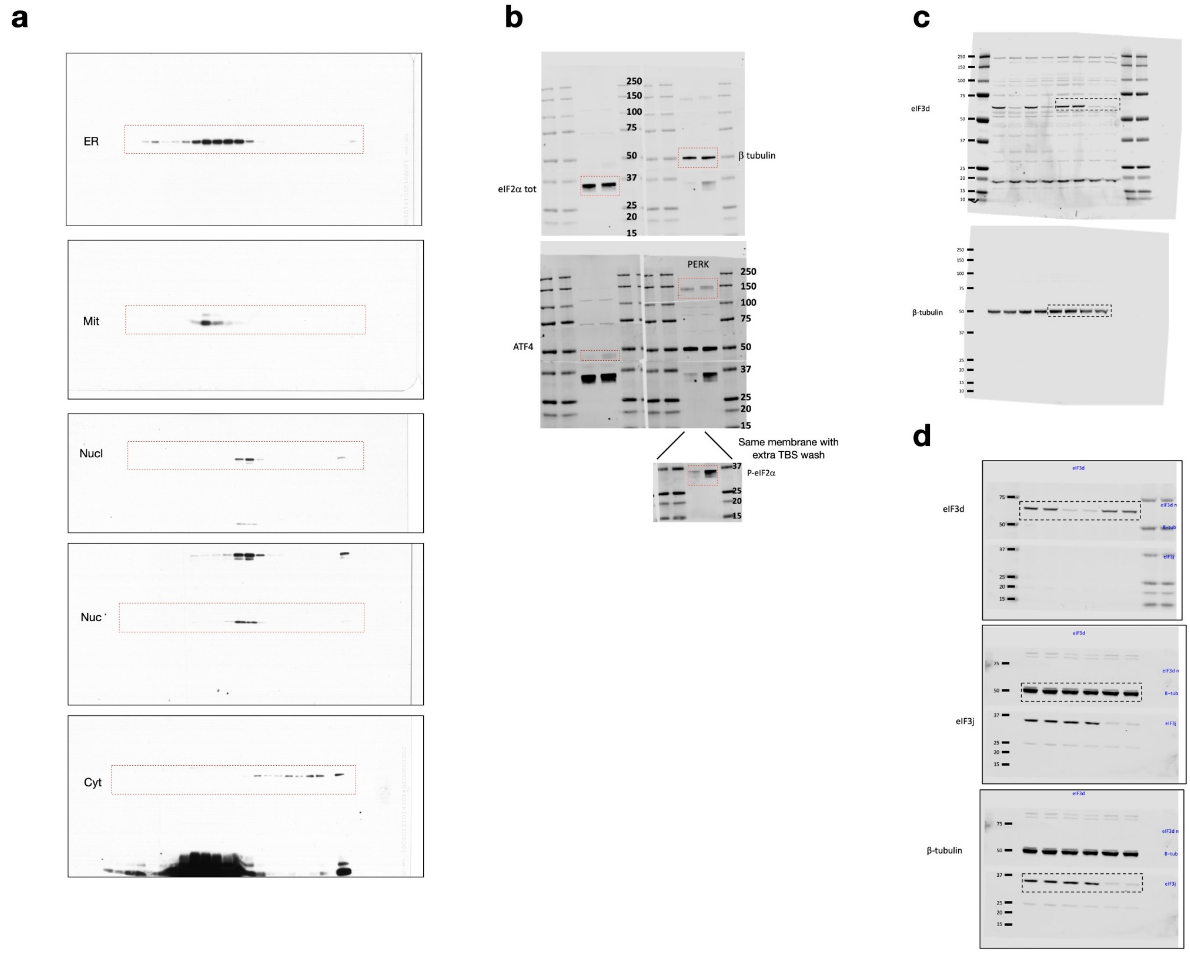

Supplementary figure 7. Uncropped western blots

a, Uncropped western blots form supplementary figure 1c. Membrane slices are highlighted in red.

b, Uncropped western blots form supplementary figure 4a. Displayed areas in main figures are highlighted in red.

c, Uncropped western blots form supplementary figure 6d. Displayed areas in main figures are highlighted in black.

d, Uncropped western blots form supplementary figure 6g. Displayed areas in main figures are highlighted in black.

Table S1. Normalised RNA abundances, related to figures 1 and 2
Tables comprise gene and transcript-level abundances for control and upr conditions for density-based LoRNA and gene-level abundances for sedimentation-based LoRNA. Column name suffixes denote replicate and fraction number. Abundances are row-sum normalised within each replicate.

Table S2. Localisation proportions, related to figures 2, 3 and 4.
Tables are included for gene and transcript-level proportions for control and upr conditions for density-based LoRNA and gene-level proportions for sedimentation-based LoRNA. Further tables list the subsets of genes whose protein product is not predicted to encode a signal peptides or transmembrane domain but have > 35% membrane localisation in control or upr conditions. Column markers denotes whether a gene/transcript was a marker for a localisation. Columns prefixed with sd_ describe the standard deviation for the localisation proportion across the 3 replicates. Column signal_or_TM denotes whether the gene/transcript is predicted to encode a signal peptides or transmembrane domain.

Table S3. Protein subcellular localisation and relocalisation upon UPR activation, related to figure 4
The table includes the predicted subcellular localisation of 5315 protein in control and UPR conditions. Column markers denote whether a protein was a marker for a localisation. Columns tsne_dim1 and tsne_dim2 denote the tSNE coordinates, as shown in supplementary figure 4J. Columns primary_localisation_per_rep and primary_localisation denote the most probable localisation per replicate and, where replicates agree, the most probable localisation for the protein. Column diff_loc_level indicates the confidence level of relocalisation events, Columns n_diff_loc_reps and diff_loc_r_reps indicate how many replicates relocalisation was observed in and which replicates, respectively.

Table S4. Experimental conditions, related to all figures.
Tables comprise: Exact p values of Fig 5g, exact p values of Fig 5c, exact p values of Fig 5d, TMT labelling schemas, gradient fraction merging schemas for RNA and protein and antibodies used.
