# Supplementary material for: System-wide analysis of RNA and protein subcellular localization dynamics
Source: Nat Methods. 2023 Nov 30;21(1):60–71. doi: 10.1038/s41592-023-02101-9 (PMC10776395; doi:10.1038/s41592-023-02101-9)
Supplement: Supplementary file 2 — Reporting Summary [file 41592_2023_2101_MOESM2_ESM.pdf]

nature portfolio

Corresponding author(s): Kathryn S Lilley and Anne E Willys

Last updated by author(s): 25-09-2023

Reporting Summary

Nature Portfolio wishes to improve the reproducibility of the work that we publish. This form provides structure for consistency and transparency in reporting. For further information on Nature Portfolio policies, see our Editorial Policies and the Editorial Policy Checklist.

Statistics

For all statistical analyses, confirm that the following items are present in the figure legend, table legend, main text, or Methods section.

n/a	Confirmed	
☐	☒	The exact sample size (*n*) for each experimental group/condition, given as a discrete number and unit of measurement
☐	☒	A statement on whether measurements were taken from distinct samples or whether the same sample was measured repeatedly
☐	☒	The statistical test(s) used AND whether they are one- or two-sided<br>*Only common tests should be described solely by name; describe more complex techniques in the Methods section.*
☐	☒	A description of all covariates tested
☐	☒	A description of any assumptions or corrections, such as tests of normality and adjustment for multiple comparisons
☐	☒	A full description of the statistical parameters including central tendency (e.g. means) or other basic estimates (e.g. regression coefficient) AND variation (e.g. standard deviation) or associated estimates of uncertainty (e.g. confidence intervals)
☐	☒	For null hypothesis testing, the test statistic (e.g. *F*, *t*, *r*) with confidence intervals, effect sizes, degrees of freedom and *P* value noted<br>*Give P values as exact values whenever suitable.*
☐	☒	For Bayesian analysis, information on the choice of priors and Markov chain Monte Carlo settings
☐	☒	For hierarchical and complex designs, identification of the appropriate level for tests and full reporting of outcomes
☐	☒	Estimates of effect sizes (e.g. Cohen's *d*, Pearson's *r*), indicating how they were calculated

Our web collection on statistics for biologists contains articles on many of the points above.

Software and code

Policy information about availability of computer code

Data collection	Confocal microscopy: Images were acquired using ZEN Blue software v3.3 and processed using FiJi software v2.3.051.
Data analysis	Proteomics data processing: Raw data were viewed in Xcalibur v.2.1. Peptide spectrum matching was performed with Proteome Discoverer v2.3 with SequestHF and MS Amanda algorithm<br><br>Transcriptomics data processing: Fastq files were demultiplexed using idemux (https://github.com/Lexogen-Tools/idemux). UMIs were extracted using UMI-tools v1.1.0. Reads were aligned using hisat v2.2.1. Quantification was performed using Salmon v1.4.0.<br><br>Data analyses was performed using R v4.0.3 and R markdown notebooks, using multiple packages, predominantly ggplot2 v3.4.2, tidyr v1.2.0, dplyr v1.0.8, MSnbase v2.20.4, pRoloc v1.34.0, camprotR v0.0.0.900 (https://github.com/CambridgeCentreForProteomics/camprotR), tximport v1.22.0, DESeq2 v1.34.0, BANDLE v1.0, ENCODExplorer v2.16.0, GenomicFeatures v1.46.5, glmnet v4.1-3 and goseq v1.46.0.<br><br>Data analysis code is available from https://github.com/CambridgeCentreForProteomics/LoRNA_UPR (v1.0) and archived with zenodo, DOI: 10.5281/zenodo.8375646 .

For manuscripts utilizing custom algorithms or software that are central to the research but not yet described in published literature, software must be made available to editors and reviewers. We strongly encourage code deposition in a community repository (e.g. GitHub). See the Nature Portfolio guidelines for submitting code & software for further information.

Data

Policy information about availability of data

All manuscripts must include a data availability statement. This statement should provide the following information, where applicable:

- Accession codes, unique identifiers, or web links for publicly available datasets
- A description of any restrictions on data availability
- For clinical datasets or third party data, please ensure that the statement adheres to our policy

The mass spectrometry proteomics data generated in this study have been deposited to the ProteomeXchange Consortium via the PRIDE partner repository with the dataset identifier PXD030456.

The RNA-Seq data generated in this study have been deposited in ENA with study accession PRJEB49479.

RNA annotations were obtained from ensembl v102 using biomaRt R package

FANTOM5 data was downloaded from https://www.nature.com/articles/s41587-021-00936-1

eCLIP and CeFra-Seq data were downloaded from https://www.encodeproject.org

The hyperLOPIT and LOPIT-DC data were obtained using R package pRolocdata (object names: hyperLOPITU2OS2018, lopitdcU2OS2018)

The following additional publicly available datasets were used. In all cases the data used is available from https://github.com/CambridgeCentreForProteomics/LoRNA_UPR (v1.0; archived with zenodo, DOI: 10.5281/zenodo.8375646) in the directory 1_external

Human Protein Atlas data was downloaded from https://www.proteinatlas.org/about/download on 03 MAY 2023

MERFISH data was downloaded from https://www.pnas.org/doi/10.1073/pnas.1912459116#data-availability

Ribosome-associated lncRNAs classification was obtained from https://www.ncbi.nlm.nih.gov/pmc/articles/PMC5975437

ER Riboseq data was obtained from https://www.science.org/doi/10.1126/science.1257521

P-body enriched RNA data was obtained from DOI: https://doi.org/10.1016/j.molcel.2017.09.003

APEX-Seq data was obtained from https://www.ncbi.nlm.nih.gov/geo/query/acc.cgi?acc=GSE116008

APEX-RIP data was obtained from https://www.ncbi.nlm.nih.gov/geo/query/acc.cgi?acc=GSE106493

IRES data was downloaded from IRESbase (http://reprod.njmu.edu.cn/cgi-bin/iresbase/download.php#human)

uORF data was downloaded from https://doi.org/10.1093/nar/gky188

GO term annotations were downloaded from ensembl v102 using biomaRt R package

Data pertaining to the effect of eIF3D Knockdown on translation was downloaded from https://doi.org/10.1016/j.molcel.2020.06.003 (Supplemental Information; Data S1)

5' cap eIF3d binding data was obtained from DOI: 10.1126/science.abb0993

Signal peptide and transmembrane domain annotations were downloaded from Uniprot (https://www.uniprot.org/) using the search string '(annotation:(type:signal) OR annotation:(type:transmem)) AND organism:"Homo sapiens (Human) [9606]"' on 06 SEP 21

Data pertaining to stress granule enrichment upon arsenite treatment were downloaded from https://doi.org/10.1016/j.molcel.2017.10.015

Data pertaining to TIS-granules were downloaded from DOI: 10.1101/2022.11.04.515216

RNA modification data was downloaded from m6A Atlas (http://180.208.58.19/m6A-Atlas/download.html) on 02 Nov 2022

Human research participants

Policy information about studies involving human research participants and Sex and Gender in Research.

Reporting on sex and gender	NA
Population characteristics	NA
Recruitment	NA

Ethics oversight | NA

Note that full information on the approval of the study protocol must also be provided in the manuscript.

Field-specific reporting

Please select the one below that is the best fit for your research. If you are not sure, read the appropriate sections before making your selection.

☒ Life sciences ☐ Behavioural & social sciences ☐ Ecological, evolutionary & environmental sciences

For a reference copy of the document with all sections, see nature.com/documents/nr-reporting-summary-flat.pdf

Life sciences study design

All studies must disclose on these points even when the disclosure is negative.

Sample size: No statistical methods were used to predetermine sample size. Sample sizes were chosen based on experience of the relevant techniques and an assessment of the observed variance.

Data exclusions: No data was excluded

Replication: Density-based LoRNA: 3 independent experiments were performed. Moreover, an orthogonal sedimentation-based LoRNA was developed which verified the accuracy of the localisation proportions estimates and ensured they were not biased by the fractionation approach. This sedimentation-based LoRNA was applied in 5 independent experiments.

Cell migration assays: 5 independent experiments were performed which verified the reproducibility of the observed effect of eIF3D knockdown.

smFISH/IF: 3 independent experiments were performed which verified the reproducibility of the observed relocalisation of RNA upon activation of the UPR.

Randomization: Cell culture plates were randomly assigned to experimental conditions.

Blinding: Blinding was not required in our experimental design since we did not allocate to groups and data processing and analysis were performed identically for all samples, precluding human bias.

Reporting for specific materials, systems and methods

We require information from authors about some types of materials, experimental systems and methods used in many studies. Here, indicate whether each material, system or method listed is relevant to your study. If you are not sure if a list item applies to your research, read the appropriate section before selecting a response.

Materials & experimental systems

n/a	Involved in the study
☐	☒ Antibodies
☐	☒ Eukaryotic cell lines
☒	☐ Palaeontology and archaeology
☒	☐ Animals and other organisms
☒	☐ Clinical data
☒	☐ Dual use research of concern

Methods

n/a	Involved in the study
☒	☐ ChIP-seq
☒	☐ Flow cytometry
☒	☐ MRI-based neuroimaging

Antibodies

Antibodies used:
Anti-Calnexin Abcam ab22595
Anti-ZNF622 Santa-Cruz sc-10098
Goat anti-Rabbit IgG Invitrogen, A-21245
Goat anti-Mouse IgG Invitrogen, A-11029
Anti-b-tubulin CST #2146
Anti-eIF3d Proteintech #66024-1-lg
Anti-fibrillarin Cell Signaling C13C3
anti-histone H3 Bethyl Laboratories #A300-823A
anti-beta actin Abcam ab8227
anti-calreticulin Cell Signaling D3E6
Anti-COX 1V Cell Signalling 4850

Validation	ab22595 Knockout and WB validated by manufacturer; sc-100980 WB validation by the manufacturer & used in PMID: 26240280; #2146 WB validation by manufacturer & used in PMID: 36869047; #66024-1-Ig WB validation by the manufacturer; C13C3 WB validation by manufacturer & used in: PMID: 34158490; #A300-823A WB validated by the manufacturer; ab8227 WB validation by manufacturer; D3E6 WB validation by the manufacturer; 4850 WB validation by the manufacturer

Eukaryotic cell lines

Policy information about cell lines and Sex and Gender in Research

Cell line source(s)	U-2 OS (U2OS) were obtained from the American Type Culture Collection (ATCC).
Authentication	None of the cells were authenticated
Mycoplasma contamination	All cells were regularly tested for mycoplasma contamination with negative results
Commonly misidentified lines (See ICLAC register)	No commonly misidentified cell lines were used in this study
